# *Neuregulin 1*: a prime candidate for research into gene-environment interactions in schizophrenia? Insights from genetic rodent models

**DOI:** 10.3389/fnbeh.2013.00106

**Published:** 2013-08-15

**Authors:** Tim Karl

**Affiliations:** ^1^Neuroscience Research AustraliaRandwick, NSW, Australia; ^2^Schizophrenia Research InstituteDarlinghurst, NSW, Australia; ^3^School of Medical Sciences, University of New South WalesNSW, Australia

**Keywords:** schizophrenia, *neuregulin 1*, gene-environment interactions, mouse, rat, stress, enrichment, housing

## Abstract

Schizophrenia is a multi-factorial disease characterized by a high heritability and environmental risk factors. In recent years, an increasing number of researchers worldwide have started investigating the “two-hit hypothesis” of schizophrenia predicting that genetic and environmental risk factors (GxE) interactively cause the development of the disorder. This work is starting to produce valuable new animal models and reveal novel insights into the pathophysiology of schizophrenia. This mini review will focus on recent advancements in the field made by challenging mutant and transgenic rodent models for the schizophrenia candidate gene *neuregulin 1* (*NRG1*) with particular environmental factors. It will outline results obtained from mouse and rat models for various Nrg1 isoforms/isoform types (e.g., transmembrane domain *Nrg1*, Type II *Nrg1*), which have been exposed to different forms of stress (acute *versus* chronic, restraint *versus* social) and housing conditions (standard laboratory *versus* minimally enriched housing). These studies suggest *Nrg1* as a prime candidate for GxE interactions in schizophrenia rodent models and that the use of rodent models will enable a better understanding of GxE interactions and the underlying mechanisms.

## Introduction

The two-hit hypothesis of schizophrenia states that a combination of genetic and environmental risk factors causes the development of schizophrenia (Bayer et al., 1999; Rapoport et al., 2005; Caspi and Moffitt, 2006). Indeed, twin studies show that nature and nuture are both important in the development of schizophrenia (i.e., concordance rate of monozygotic twins is 50%) (Tsuang et al., 2001) and combined actions of multiple genes of small effect (Owen et al., 2005) and a number of environmental risk factors (McGrath et al., 2004) is likely (Mackay-Sim et al., 2004). Genome wide association studies suggest that it is important to consider the interplay of genes and environment to understand the aetiology of the disorder in more depth (Sanders et al., 2008). In this context, interactions of genetic and environmental risk factors (GxE) occur when an individual's genetic predispositions are expressed dependent on their environment or when environmental influences on a trait differ according to the individual's genome (Tsuang et al., 2004). According to the neurodevelopmental theory of schizophrenia genes and environment together affect brain development negatively during critical periods of neuronal development and thereby induce schizophrenia (Marenco and Weinberger, 2000).

Animal models can incorporate genetic and environmental risk factors at different stages of development, thereby more accurately mimicking the aetiology of schizophrenia, and help elucidate interactions between those factors and underlying mechanism (Burrows et al., 2011). For example, *neuregulin 1* (*NRG1*) is a genetic target for schizophrenia research (Stefansson et al., 2002; Tosato et al., 2005; Munafo et al., 2006) as it influences key neurodevelopmental processes relevant to schizophrenia (e.g., myelination and neuronal migration), and regulates receptors such as N-methyl-D-aspartic acid (NMDA) and γ-aminobutyric acid receptor A (GABA_A_) (Mei and Xiong, 2008). It has been outlined that there might be genetic subgroups in the population that are more vulnerable to particular environmental risk factors (e.g., cannabis abuse, developmental trauma) (van Os et al., 2010) and *NRG1* might be such a genetic candidate. This review will summarize preclinical data to assess if *Nrg1* might be mediating an increased risk to environmental factors with relevance to schizophrenia (i.e., stress and cannabis) and experimental animal research (i.e., laboratory housing conditions).

## Nrg1 X laboratory housing

Environmental enrichment (EE) has a significant impact on animal models of neurodegenerative diseases (van Dellen et al., 2000; Spires and Hannan, 2005). Furthermore, enriched cage structures can modify or even rescue knockout-specific abnormalities of genetic mouse models (Rampon et al., 2000; van Dellen et al., 2000). Thus, the behavioral effects of minimally enriched housing (ME) compared to standard laboratory housing were determined in male transmembrane domain *Nrg1* mutant and wild type-like control mice (Karl et al., 2007). This mutant mouse model has been shown to have compelling construct, face, and predictive validity for schizophrenia (Stefansson et al., 2002; Walss-Bass et al., 2006; Karl et al., 2007, 2011; van den Buuse et al., 2009; Duffy et al., 2010; Chesworth et al., 2012a). Mice were tested at the age of 3–4 and 4–6 months, as the age of patients has a significant impact on the aetiology of schizophrenia (Thompson et al., 2004). Effects of *Nrg1* mutation on locomotion, exploration and anxiety-like behaviors were age-dependent and interacted with the housing condition males were raised in. *Nrg1* mutants kept in ME developed hyper-exploration in the light-dark test and reduced anxiety-like behavior in the open field test at 3–4 months of age whereas *Nrg1* males kept in standard housing displayed these phenotypes only at the age of 4–6 months. Importantly, well-known explorative and anxiolytic-like properties of cage enrichment (Chapillon et al., 1999; Roy et al., 2001; Benaroya-Milshtein et al., 2004) were more pronounced in *Nrg1* mutant mice than control mice suggesting that mutant transmembrane domain *Nrg1* increased the behavioral sensitivity to ME.

*Nrg1* mutant mice are characterized by hypo-phosphorylation of the NR2B subunit (Bjarnadottir et al., 2007). This is in line with Nrg1's up-regulation of NMDA subunits expression (Ozaki et al., 1997; Stefansson et al., 2004) and the stimulation of Y1472 phosphorylation on the NR2B subunit of NMDA receptors. As NMDA antagonists induce increased locomotor activity (Wong and Van Tol, 2003; Javitt and Coyle, 2004) and as mouse models for NMDA receptors suggest an involvement of the glutamatergic system in rodent hyperactivity (Smith et al., 1998; Dulawa et al., 1999; Mohn et al., 1999; Zhuang et al., 2001), hypo-phosphorylated NR2 subunits may be responsible for the observed hyperactivity. Nonetheless, it should be noted that Hahn and colleagues found that Nrg1 stimulation suppressed NMDA receptor activation in the human prefrontal cortex (Hahn et al., 2006). EE does not impact the behavioral susceptibility to NMDA receptor antagonists, but mRNA expression of specific NMDA receptor subunits was decreased in mice kept in enriched housing (Grilli et al., 2009). This suggests that combined effects of mutant *Nrg1* and ME (i.e., additive GxE) might be responsible for the earlier onset of hyperactivity.

Importantly, hypo-phosphorylation of NR2B subunits in *Nrg1* mutant mice might also support the activation of dopaminergic pathways (Duncan et al., 1999; Kapur and Seeman, 2002) and thereby contribute to their anxiolytic-like and hyper-locomotive phenotype. Indeed, dopamine transporter deficient mice are not only characterized by hyperactivity but also decreased anxiety-like-like responses (Carpenter et al., 2012). In this context, it is important to note that exposure to enriched housing affects the dopaminergic system as enrichment increased the susceptibility of rats to the behavioral and neurochemical effects of amphetamine (Bowling et al., 1993) although another study found reduced dopamine receptor 1 function as a consequence of enriched housing (Del Arco et al., 2007). Further research is needed to pinpoint the mechanisms underlying the differential potency of ME in *Nrg1* mutant and control mice but an involvement of dopaminergic and glutamatergic circuits is likely.

Other genetic mouse models of schizophrenia have been reported to benefit from more complex enriched housing environments (McOmish et al., 2008). Thus, the disease-related phenotype-strengthening effects of ME in *Nrg1* mice are interesting and opposite to reports by others (van Dellen et al., 2000; Olsson and Dahlborn, 2002; Spires and Hannan, 2005). *Nrg1/NRG1* has been described as being critical for how an organism responds and adapts to the environment (Stefansson et al., 2004). Thus, the biological function of *Nrg1* may dictate this disease phenotype-strengthening response to an enriched housing environment, which is different to the effects normally described for EE (i.e., reversing disease phenotypes).

## Nrg1 X stress

Stressful life events and changes in HPA axis function are associated with and precipitate the onset of psychiatric disorders (Koenig et al., 2002; Walker et al., 2008). Furthermore, stress plays a role in the development [e.g., behavioral sensitization: (van Os et al., 2010)] and severity of psychotic symptoms (Corcoran et al., 2003) and triggers relapse in schizophrenia patients (Hultman et al., 1997). There appears to be a genetic component to stress vulnerability in schizophrenia: schizophrenia patients are more sensitive to stress (van Winkel et al., 2008), handle negative life events more poorly (Horan et al., 2005), and show impaired cortisol and HPA axis activity in stressful situations (van Venrooij et al., 2010). Importantly, a *NRG1* polymorphism interacts with psychosocial stress thereby affecting reactivity to expressed emotions in schizophrenia patients (Keri et al., 2009) and *NRG1* also interacts with job strain thereby increasing the risk of heart disease (Hintsanen et al., 2007). Furthermore, Nrg1 is expressed in brain regions controlling stress reactivity (Chen, 2007). Thus, a number of research teams have investigated the response of *Nrg1* rodent models to models.

A first study investigated the behavioral and endocrine response of male transmembrane domain *Nrg1* mutant mice to acute restraint stress before and after the onset of the age-dependent hyper-locomotive phenotype (Chesworth et al., 2012b). The suppressive effect of stress on locomotion was evident in all mice regardless of genotype or age. Surprisingly, older *Nrg1* mutants appeared insensitive to anxiety-like-related stress effects in the open field (i.e., center locomotion). All mice displayed robust stress-induced increases in serum corticosterone, although the response was more pronounced in young *Nrg1* mutants compared to WT mice. The study suggested that there is no pronounced effect of mutant transmembrane domain *Nrg1* on the endocrine and behavioral effects of acute restraint stress. Nevertheless, Nrg1 modified corticosterone release in young *Nrg1* mutants and the anxiety-like response of hyper-locomotive older *Nrg1* mice, confirming that the gene plays a role in how an organism responds to environmental manipulations. The phenomenon of a disconnected behavioral and endocrine stress response of older *Nrg1* mice (i.e., no stress-induced anxiety-like response in open field but increased glucocorticoid levels) is consistent with other mouse models (Laarakker et al., 2011; Trainor et al., 2011). Future research should address the impact of chronic stress on *Nrg1* mutant mice and consider additional aspects of HPA functions.

Importantly, recent rat research suggests that *Nrg1* might be involved in stress reactivity downstream from the release of glucocorticoids (Taylor et al., 2011b). More specifically, a rat model for disrupted Type II *Nrg1* expression was characterized by increased baseline corticosterone levels and improved recovery of corticosterone levels post-acute restraint stress. Importantly, in control rats, Type II *Nrg1* was expressed in the neurocircuitry involved in regulating HPA responses to environmental stimuli. The authors concluded that disruptions to Type II *Nrg1* expression mediated an increased basal HPA axis activity. Elevated levels of glucocorticoid (but not mineralocorticoid) receptors in the hippocampus and pituitary glands of *Nrg1* mutant rats under baseline conditions could then result in a more pronounced negative feedback loop thereby increasing the inhibition of HPA axis activity following acute restraint stress. Interestingly, shifts in the balance of glucocorticoid and mineralocorticoid receptor levels in humans can create a vulnerability to psychiatric disease, especially among genetically predisposed individuals (De Kloet et al., 1998; Zhe et al., 2008). The change in the endocrine stress response of mutant Type II rats was accompanied by altered habituation to an open field environment across test days (Taylor et al., 2011b). Nrg1 is necessary for the establishment of excitatory synapses in GABAergic interneurons and for the development of a balanced excitatory/inhibitory tone in the brain (Ting et al., 2011). As GABAergic mechanisms play a role in controlling HPA axis function (Herman et al., 2004), Nrg1-induced changes to the GABAergic system might present a potential mechanism for the observations in Type II *Nrg1* mutant rats.

In a follow-up study it was found that some of the behavioral and brain characteristics of *Nrg1* hypomorphic rats were highly sex-specific (Taylor et al., 2011a). It should be noted that sex-specificity in rodent models for *Nrg1* is a common phenomenon (O'Tuathaigh et al., 2006; Duffy et al., 2010; Chesworth et al., 2012a) and is in line with gender effects reported for schizophrenia patients (Canuso and Pandina, 2007). Inconsistencies between the stress response of the two investigated *Nrg1* rodent models are most likely due to (1) species differences (Asan et al., 2005), (2) differences in the restraint stress models used (rats were habituated to the general stress procedure whereas mice were naïve), and (3) the particular characteristics of the *Nrg1* mutation [(Harrison and Law, 2006; Mei and Xiong, 2008); for overview on *Nrg1* rodent models see: (Duffy et al., 2008; Karl et al., 2011)]. Adding to the complexity of potential *Nrg1*-stress interactions is a study reporting that Type III *Nrg1* mutant mice display a blunted increase in corticosterone release after mild acute stress (Chen, 2007).

Adolescence is a period of heightened risk to develop schizophrenia (Walker and Bollini, 2002; Costello et al., 2003; Paus et al., 2008) as abnormal adolescent brain development contributes to the aetiology of schizophrenia (Paus et al., 2008; Walker et al., 2008). Furthermore, stress response-relevant neuronal pathways develop during adolescence (Andersen et al., 2000; Spear, 2000; Casey et al., 2008) and HPA axis plasticity appears sensitive to adolescent stress exposure as well (Romeo et al., 2006). Thus, it is important to assess interactions between Nrg1 and stress also during adolescence.

Indeed, Taylor and co-workers investigated the effects of chronic variable stress during adolescence on endocrine and behavioral measures in adult Type II *Nrg1* mutant rats (Taylor et al., 2012). Sex-specific interactions between *Nrg1* genotype and adolescent stress were found. Stress during adolescence reduced baseline corticosterone levels in female control but not mutant rats. Furthermore, stress increased extinction of cued fear conditioning but only in *Nrg1* females. The authors concluded that the findings represent a true *Nrg1* x stress interaction and are consistent with a reduction in sensitivity to environmental stimuli and novelty as described earlier (Taylor et al., 2011a,b). However, *Nrg1* females were the only group susceptible to the effects of adolescent stress on fear extinction. In addition, most earlier findings had been evident in male rats (Taylor et al., 2011b), which failed to be affected by the adolescent stress model chosen.

Social defeat stress models aspect of psychosocial stress in humans, which has been found to interact with a single nucleotide polymorphism of *NRG1* to affect the reactivity of schizophrenia patients to expressed emotion (Keri et al., 2009). Psychosocial stress might also contribute to the development of schizophrenia via sensitization of the pro-inflammatory immune response leading to excessive pro-inflammatory cytokine release. Thus, researchers investigated behavioral and neuro-physiological effects of adolescent repeated intermittent social defeat in adult transmembrane domain *Nrg1* mutant males (Desbonnet et al., 2012) and found that *Nrg1* modified the effects of social defeat on several behavioral, immunological and brain measures. For example, psychosocial stress diminished the hyper-locomotive phenotype of *Nrg1* mutant mice without accompanying effects on control littermates. In addition, stress had cognitive-impairing effects in *Nrg1* mice only and decreased sucrose preference (model for anhedonia) in control but not mutant mice. Social defeat also altered the lipopolysaccharide and concanavalin A-stimulated cytokine response of the spleen in a genotype-specific manner (see study for details). In the brain, stress decreased interleukin-beta mRNA levels in the prefrontal cortex of mutant mice only, whereas striatal interleukin-beta was down-regulated in controls and up-regulated in *Nrg1* mice. Finally, hippocampal BDNF mRNA levels were elevated in control mice and reduced in mutant mice whereas tumor necrosis factor-alpha was up-regulated in *Nrg1* mice only. Reduced striatal BDNF levels might have been involved in the disrupting effects of social defeat stress on the spatial memory of *Nrg1* mutant mice (Almli et al., 2000). Importantly, Nrg1 can interact with BDNF in regulating neuronal processes (Mei and Xiong, 2008; Balu and Coyle, 2011), BDNF down-regulation has been reported in schizophrenia (Weickert et al., 2003; Favalli et al., 2012), and BDNF expression changes impact on the sensitivity to social defeat stress (Berton et al., 2006; Krishnan et al., 2007). The authors concluded that the experience of psychosocial stress during adolescence may trigger further pathophysiological features that contribute to the development of schizophrenia in individuals underlying *NRG1* gene abnormalities. The interactive nature of the effects of stress and mutant *Nrg1* resulted in cognitive deficits and an imbalance in BDNF and immunological parameters. On the other side, stress impacted positively on the hyper-locomotive phenotype of *Nrg1* mutant mice, outlining the complexity of GxE interactions in schizophrenia and the need to look at specific disease endophenotypes.

In summary, research teams have started evaluating the role of *Nrg1* in the neuro-endocrine, behavioral, and immunological response of mice to stress. Results so far are inconclusive demanding that future research should focus on schizophrenia-relevant stress models [similar to (Desbonnet et al., 2012)], consider sex and age in experimental designs, and focus on schizophrenia-like behaviors and disease-relevant brain markers.

## Nrg1 X cannabis

A review on the role of *Nrg1* in GxE in schizophrenia would be incomplete without mentioning the extensive mouse work on *Nrg1* x cannabis interactions. As those studies have been reviewed elsewhere (Arnold et al., 2012; Karl and Arnold, 2013; Ng et al., 2013), this section will only provide a brief summary. It has long been established that cannabis is a component/cumulative cause for the development of schizophrenia (Arseneault et al., 2002, 2004) suggesting interactions with other risk factors (D'Souza et al., 2009). Until recently, Catechol-O-methyltransferase (*COMT*) was the only candidate for a possible interaction between a genetic predisposition for schizophrenia and heavy cannabis abuse [(Caspi et al., 2005; O'Tuathaigh et al., 2010) but see also (Zammit et al., 2011)]. Comprehensive analyses on *Nrg1* x cannabis interactions in transmembrane domain *Nrg1* mutant mice suggest that *Nrg1* increases the susceptibility of an organism to the neuro-behavioral effects of cannabis as well (Boucher et al., 2007a,b, 2011; Long et al., 2010, 2012, 2010). The clinical relevance of this research has recently been highlighted by a genetic study in African Americans, which discovered *NRG1* as a major candidate for the development of cannabis dependence (Han et al., 2012).

## Conclusions

Recent research utilizing genetic rodent models has revealed an interactive relationship between *Nrg1* and a variety of environmental factors. These interactions appear to be complex and sensitive to a number of subtle variables, but do exist and justify the need for future research in this area (van Os et al., 2010). Researchers should focus on models with significant relevance to schizophrenia including, for example, cannabis abuse (discussed above) and maternal immunization (Ibi et al., 2010; Giovanoli et al., 2013) and consider not only *Nrg1* but also other genetic candidates for GxE interactions. Importantly, the research into *Nrg1*xE outlined above suggests that valid GxE mouse models will be very sensitive to the laboratory environment and other potential test confounders (e.g., age and sex) so that a high level of transparency and standardization of test conditions across research sites will be crucial.

Although the exact nature of *Nrg1*xE and their consequences for schizophrenia have to be evaluated further, an involvement of the GABAergic, glutamatergic and BDNF systems seems likely. Importantly, environmental (risk) factors not always induced adverse (i.e., disease phenotype-strengthening) effects in *Nrg1* mutants, which should be taken into account when looking into GxE interactions [for genotype-specific effects of environmental factors see also (Tucci et al., 2006; Valdar et al., 2006)]. The findings on *Nrg1*xE summarized in Table 1 are in line with the GxE theory, contribute to the understanding of the pathogenesis of schizophrenia, and might eventually help with possible early intervention programs. Importantly, recent discussions on the appropriate statistical modeling of GxE interactions (van Winkel et al., 2008; Zammit et al., 2010) as well as the limitations of animal model research into schizophrenia (Ayhan et al., 2009) should be considered for future work.

**Table 1:** **Effects of environmental factors on rodent models for the schizophrenia candidate gene *neuregulin 1***.

***Nrg1* × Laboratory housing (i.e., minimal enrichment)**	
Transmembrane domain *Nrg1* mutant male mice (Karl et al., 2007)	Minimal enrichment shifted the onset of the hyper-explorative and anxiogenic phenotype of *Nrg1* mice to 3–4 months of age compared to mutant mice kept in standard laboratory housing (onset at 4–6 months of age).
***Nrg1* × Stress**	
Acute restraint stress	No pronounced effect of *Nrg1* on the endocrine and behavioral effects of acute restraint stress—only subtle, age-dependent modification of stress-induced corticosterone release and anxiety-like behaviors.
Transmembrane domain *Nrg1* mutant male mice (Chesworth et al., 2012b)	
Acute restraint stress	Altered habituation to an open field environment in *Nrg1* mutant rats.
Adult Type II *Nrg1* mutant rats (Taylor et al., 2011a,b)	Mutant *Nrg1* resulted in increased baseline corticosterone levels and improved recovery of those levels post stress.
	Elevated baseline levels of glucocorticoid receptors in hippocampus and pituitary glands. Results are highly sex-specific.
Chronic variable stress	Female *Nrg1* rats displayed no stress-induced reduction in corticosterone levels and showed increased extinction of cued fear conditioning (no such effects in male *Nrg1* mutants).
Adolescent Type II *Nrg1* mutant rats (Taylor et al., 2012)	
Social defeat stress	Stress diminished hyper-locomotion and induced cognitive deficits in *Nrg1* mutant mice without accompanying effects in control mice. *Nrg1* mutant mice were protected against anhedonic properties of social defeat.
Transmembrane domain *Nrg1* mutant mice (Desbonnet et al., 2012)	
	The effects of stress on the cytokine response of mice were genotype-dependent (for details see study).
	Stress decreased interleukin-beta mRNA levels in the prefrontal cortex of *Nrg1* mice. Striatal interleukin-beta levels were reduced in control mice and increased in *Nrg1* mice. Hippocampal BDNF mRNA levels were elevated in control mice and reduced in mutant mice whereas tumor necrosis factor-alpha was up-regulated in *Nrg1* mice only.
***Nrg1* × Cannabis reviewed in (Arnold et al., 2012; Karl and Arnold, 2013; Ng et al., 2013)**	
Acute treatment with Δ ^9^-tetrahydrocannabinol (THC)	*Nrg1* mutants displayed a sex-dependent increased susceptibility to the locomotion-suppressive effects of THC and showed improved prepulse inhibition post THC treatment.
Adult transmembrane domain *Nrg1* mutant mice (Boucher et al., 2007a,b; Long et al., 2010)	
	THC induced increased neuronal activity in the ventral part of the lateral septum and greater activity in central nucleus of the amygdala and the paraventricular nucleus of the hypothalamus in *Nrg1* mutant mice.
Chronic treatment with CP55,940 (CP)	*Nrg1* mutants developed a behavioral tolerance to CP-induced hypothermia and hypolocomotion more rapidly, whereas the same mice did not develop a tolerance to CPs anxiogenic effects.
Adult transmembrane domain *Nrg1* mutant male mice (Boucher et al., 2011)	
	Mutant mice showed a selectively increase in CP-induced FosB/Δ FosB expression in the ventral part of lateral septum.
Chronic THC treatment	THC exacerbated hyperlocomotion 48 h after THC withdrawal. *Nrg1* mutant mice were more resistant to social withdrawal effects of THC.
Adolescent transmembrane domain *Nrg1* mutant male mice (Long et al., 2013)	
	THC promoted genotype-dependent effects on CB1, 5-HT2A and NMDA receptor expression (see study for details).

### Conflict of interest statement

The author declares that the research was conducted in the absence of any commercial or financial relationships that could be construed as a potential conflict of interest.

## References

[B1] Almli C. R. Levy T. J. Han B. H. Shah A. R. Gidday J. M. Holtzman D. M. (2000). BDNF protects against spatial memory deficits following neonatal hypoxia-ischemia. Exp. Neurol. 166, 99–114 10.1006/exnr.2000.7492 11031087

[B2] Andersen S. L. Thompson A. T. Rutstein M. Hostetter J. C. Teicher M. H. (2000). Dopamine receptor pruning in prefrontal cortex during the periadolescent period in rats. Synapse 37, 167–169 10881038 10.1002/1098-2396(200008)37:2<167::AID-SYN11>3.0.CO;2-B

[B3] Arnold J. C. Boucher A. A. Karl T. (2012). The yin and yang of cannabis-induced psychosis: the actions of Delta(9)-tetrahydrocannabinol and cannabidiol in rodent models of schizophrenia. Curr. Pharm. Des. 18, 5113–5130 10.2174/138161212802884726 22716133

[B4] Arseneault L. Cannon M. Poulton R. Murray R. Caspi A. Moffitt T. E. (2002). Cannabis use in adolescence and risk for adult psychosis: longitudinal prospective study. BMJ 325, 1212–1213 10.1136/bmj.325.7374.1212 12446537 PMC135493

[B5] Arseneault L. Cannon M. Witton J. Murray R. M. (2004). Causal association between cannabis and psychosis: examination of the evidence. Br. J. Psychiatry 184, 110–117 10.1192/bjp.184.2.110 14754822

[B6] Asan E. Yilmazer-Hanke D. M. Eliava M. Hantsch M. Lesch K. P. Schmitt A. (2005). The corticotropin-releasing factor (CRF)-system and monoaminergic afferents in the central amygdala: investigations in different mouse strains and comparison with the rat. Neuroscience 131, 953–967 10.1016/j.neuroscience.2004.11.040 15749348

[B7] Ayhan Y. Sawa A. Ross C. A. Pletnikov M. V. (2009). Animal models of gene-environment interactions in schizophrenia. Behav. Brain Res. 204, 274–281 10.1016/j.bbr.2009.04.010 19379776 PMC2735613

[B8] Balu D. T. Coyle J. T. (2011). Neuroplasticity signaling pathways linked to the pathophysiology of schizophrenia. Neurosci. Biobehav. Rev. 35, 848–870 10.1016/j.neubiorev.2010.10.005 20951727 PMC3005823

[B9] Bayer T. A. Falkai P. Maier W. (1999). Genetic and non-genetic vulnerability factors in schizophrenia: the basis of the “two hit hypothesis.” J. Psychiatr. Res. 33, 543–548 10.1016/S0022-3956(99)00039-4 10628531

[B10] Benaroya-Milshtein N. Hollander N. Apter A. Kukulansky T. Raz N. Wilf A. (2004). Environmental enrichment in mice decreases anxiety-like, attenuates stress responses and enhances natural killer cell activity. Eur. J. Neurosci. 20, 1341–1347 10.1111/j.1460-9568.2004.03587.x 15341605

[B11] Berton O. McClung C. A. Dileone R. J. Krishnan V. Renthal W. Russo S. J. (2006). Essential role of BDNF in the mesolimbic dopamine pathway in social defeat stress. Science 311, 864–868 10.1126/science.1120972 16469931

[B12] Bjarnadottir M. Misner D. L. Haverfield-Gross S. Bruun S. Helgason V. G. Stefansson H. (2007). Neuregulin1 (NRG1) signaling through Fyn modulates NMDA receptor phosphorylation: differential synaptic function in NRG1+/- knock-outs compared with wild-type mice. J. Neurosci. 27, 4519–4529 10.1523/JNEUROSCI.4314-06.2007 17460065 PMC6672983

[B13] Boucher A. A. Arnold J. C. Duffy L. Schofield P. R. Micheau J. Karl T. (2007a). Heterozygous neuregulin 1 mice are more sensitive to the behavioural effects of Delta9-tetrahydrocannabinol. Psychopharmacology (Berl.) 192, 325–336 10.1007/s00213-007-0721-3 17333138

[B14] Boucher A. A. Hunt G. E. Karl T. Micheau J. McGregor I. S. Arnold J. C. (2007b). Heterozygous neuregulin 1 mice display greater baseline and Delta(9)-tetrahydrocannabinol-induced c-Fos expression. Neuroscience 149, 861–870 10.1016/j.neuroscience.2007.08.020 17905522

[B15] Boucher A. A. Hunt G. E. Micheau J. Huang X. McGregor I. S. Karl T. (2011). The schizophrenia susceptibility gene neuregulin 1 modulates tolerance to the effects of cannabinoids. Int. J. Neuropsychopharmacol. 14, 631–643 10.1017/S146114571000091X 20701826

[B16] Bowling S. L. Rowlett J. K. Bardo M. T. (1993). The effect of environmental enrichment on amphetamine-stimulated locomotor activity, dopamine synthesis and dopamine release. Neuropharmacology 32, 885–893 10.1016/0028-3908(93)90144-R 8232791

[B17] Burrows E. L. McOmish C. E. Hannan A. J. (2011). Gene-environment interactions and construct validity in preclinical models of psychiatric disorders. Prog. Neuropsychopharmacol. Biol. Psychiatry 35, 1376–1382 10.1016/j.pnpbp.2010.12.011 21168465

[B18] Canuso C. M. Pandina G. (2007). Gender and schizophrenia. Psychopharmacol. Bull. 40, 178–190 18227787

[B19] Carpenter A. C. Saborido T. P. Stanwood G. D. (2012). Development of hyperactivity and anxiety-like responses in dopamine transporter-deficient mice. Dev. Neurosci. 34, 250–257 10.1159/000336824 22572477

[B20] Casey B. J. Getz S. Galvan A. (2008). The adolescent brain. Dev. Rev. 28, 62–77 10.1016/j.dr.2007.08.003 18688292 PMC2500212

[B21] Caspi A. Moffitt T. E. (2006). Gene-environment interactions in psychiatry: joining forces with neuroscience. Nat. Rev. Neurosci. 7, 583–590 10.1038/nrn1925 16791147

[B22] Caspi A. Moffitt T. E. Cannon M. McClay J. Murray R. Harrington H. (2005). Moderation of the effect of adolescent-onset cannabis use on adult psychosis by a functional polymorphism in the catechol-O-methyltransferase gene: longitudinal evidence of a gene X environment interaction. Biol. Psychiatry 57, 1117–1127 10.1016/j.biopsych.2005.01.026 15866551

[B23] Chapillon P. Manneche C. Belzung C. Caston J. (1999). Rearing environmental enrichment in two inbred strains of mice: 1. Effects on emotional reactivity. Behav. Genet. 29, 41–46 10.1023/A:1021437905913 10371757

[B24] Chen Y.-J. (2007). Type III Neuregulin 1 Functions in the Central Nervous System. Ph.D. thesis, Columbia: Columbia University

[B25] Chesworth R. Downey L. Logge W. Killcross S. Karl T. (2012a). Cognition in female transmembrane domain neuregulin 1 mutant mice. Behav. Brain Res. 226, 218–223 10.1016/j.bbr.2011.09.019 21944941

[B26] Chesworth R. Yulyaningsih E. Cappas E. Arnold J. Sainsbury A. Karl T. (2012b). The response of neuregulin 1 mutant mice to acute restraint stress. Neurosci. Lett. 515, 82–86 10.1016/j.neulet.2012.03.024 22450046

[B27] Corcoran C. Walker E. Huot R. Mittal V. Tessner K. Kestler L. (2003). The stress cascade and schizophrenia: etiology and onset. Schizophr. Bull. 29, 671–692 10.1093/oxfordjournals.schbul.a007038 14989406

[B28] Costello E. J. Mustillo S. Erkanli A. Keeler G. Angold A. (2003). Prevalence and development of psychiatric disorders in childhood and adolescence. Arch. Gen. Psychiatry 60, 837–844 10.1001/archpsyc.60.8.837 12912767

[B29] D'Souza D. C. Sewell R. A. Ranganathan M. (2009). Cannabis and psychosis/schizophrenia: human studies. Eur. Arch. Psychiatry Clin. Neurosci. 259, 413–431 10.1007/s00406-009-0024-2 19609589 PMC2864503

[B30] De Kloet E. R. Vreugdenhil E. Oitzl M. S. Joels M. (1998). Brain corticosteroid receptor balance in health and disease. Endocr. Rev. 19, 269–301 10.1210/er.19.3.269 9626555

[B31] Del Arco A. Segovia G. Canales J. J. Garrido P. de Blas M. Garcia-Verdugo J. M. (2007). Environmental enrichment reduces the function of D1 dopamine receptors in the prefrontal cortex of the rat. J. Neural Transm. 114, 43–48 10.1007/s00702-006-0565-8 16955373

[B32] Desbonnet L. O'Tuathaigh C. Clarke G. O'Leary C. Petit E. Clarke N. (2012). Phenotypic effects of repeated psychosocial stress during adolescence in mice mutant for the schizophrenia risk gene neuregulin-1: a putative model of gene x environment interaction. Brain Behav. Immun. 26, 660–671 10.1016/j.bbi.2012.02.010 22426432

[B33] Duffy L. Cappas E. Lai D. Boucher A. A. Karl T. (2010). Cognition in transmembrane domain neuregulin 1 mutant mice. Neuroscience 170, 800–807 10.1016/j.neuroscience.2010.07.042 20678553

[B34] Duffy L. Cappas E. Scimone A. Schofield P. R. Karl T. (2008). Behavioral profile of a heterozygous mutant mouse model for EGF-like domain neuregulin 1. Behav. Neurosci. 122, 748–759 10.1037/0735-7044.122.4.748 18729627

[B35] Dulawa S. C. Grandy D. K. Low M. J. Paulus M. P. Geyer M. A. (1999). Dopamine D4 receptor-knock-out mice exhibit reduced exploration of novel stimuli. J. Neurosci. 19, 9550–9556 10531457 10.1523/JNEUROSCI.19-21-09550.1999 PMC6782928

[B36a] Duncan G. E. Sheitman B. B. Lieberman J. A. (1999). An integrated view of pathophysiological models of schizophrenia. Brain Res. Brain Res. Rev. 29, 250–264 10.1016/S0165-0173(99)00002-8 10209235

[B36] Favalli G. Li J. Belmonte-de-Abreu P. Wong A. H. Daskalakis Z. J. (2012). The role of BDNF in the pathophysiology and treatment of schizophrenia. J. Psychiatr. Res. 46, 1–11 10.1016/j.jpsychires.2011.09.022 22030467

[B37] Giovanoli S. Engler H. Engler A. Richetto J. Voget M. Willi R. (2013). Stress in puberty unmasks latent neuropathological consequences of prenatal immune activation in mice. Science 339, 1095–1099 10.1126/science.1228261 23449593

[B38] Grilli M. Zappettini S. Zanardi A. Lagomarsino F. Pittaluga A. Zoli M. (2009). Exposure to an enriched environment selectively increases the functional response of the pre-synaptic NMDA receptors which modulate noradrenaline release in mouse hippocampus. J. Neurochem. 110, 1598–1606 10.1111/j.1471-4159.2009.06265.x 19723266

[B39] Hahn C. G. Wang H. Y. Cho D. S. Talbot K. Gur R. E. Berrettini W. H. (2006). Altered neuregulin 1-erbB4 signaling contributes to NMDA receptor hypofunction in schizophrenia. Nat. Med. 12, 824–828 10.1038/nm1418 16767099

[B40] Han S. Yang B. Z. Kranzler H. R. Oslin D. Anton R. Farrer L. A. (2012). Linkage analysis followed by association show NRG1 associated with Cannabis dependence in African Americans. Biol. Psychiatry 72, 637–644 10.1016/j.biopsych.2012.02.038 22520967 PMC3699339

[B41] Harrison P. J. Law A. J. (2006). Neuregulin 1 and schizophrenia: genetics, gene expression, and neurobiology. Biol. Psychiatry 60, 132–140 10.1016/j.biopsych.2005.11.002 16442083

[B42] Herman J. P. Mueller N. K. Figueiredo H. (2004). Role of GABA and glutamate circuitry in hypothalamo-pituitary-adrenocortical stress integration. Ann. N.Y. Acad. Sci. 1018, 35–45 10.1196/annals.1296.004 15240350

[B43] Hintsanen M. Elovainio M. Puttonen S. Kivimaki M. Raitakari O. T. Lehtimaki T. (2007). Neuregulin-1 genotype moderates the association between job strain and early atherosclerosis in young men. Ann. Behav. Med. 33, 148–155 10.1007/BF02879896 17447867

[B44] Horan W. P. Ventura J. Nuechterlein K. H. Subotnik K. L. Hwang S. S. Mintz J. (2005). Stressful life events in recent-onset schizophrenia: reduced frequencies and altered subjective appraisals. Schizophr. Res. 75, 363–374 10.1016/j.schres.2004.07.019 15885527

[B45] Hultman C. M. Wieselgren I. M. Ohman A. (1997). Relationships between social support, social coping and life events in the relapse of schizophrenic patients. Scand. J. Psychol. 38, 3–13 10.1111/1467-9450.00002 9104101

[B46] Ibi D. Nagai T. Koike H. Kitahara Y. Mizoguchi H. Niwa M. (2010). Combined effect of neonatal immune activation and mutant DISC1 on phenotypic changes in adulthood. Behav. Brain Res. 206, 32–37 10.1016/j.bbr.2009.08.027 19716847 PMC2846597

[B47] Javitt D. C. Coyle J. T. (2004). Decoding schizophrenia. Sci. Am. 290, 48–55 10.1038/scientificamerican0104-48 14682038

[B48] Kapur S. Seeman P. (2002). NMDA receptor antagonists ketamine and PCP have direct effects on the dopamine D(2) and serotonin 5-HT(2)receptors-implications for models of schizophrenia. Mol. Psychiatry 7, 837–844 10.1038/sj.mp.4001093 12232776

[B49] Karl T. Arnold J. C. (2013). What does a mouse tell us about neuregulin 1-cannabis interactions? Front. Cell. Neurosci. 7:18 10.3389/fncel.2013.00018 23447438 PMC3581817

[B50] Karl T. Burne T. H. Van den Buuse M. Chesworth R. (2011). Do transmembrane domain neuregulin 1 mutant mice exhibit a reliable sensorimotor gating deficit? Behav. Brain Res. 223, 336–341 10.1016/j.bbr.2011.04.051 21605597

[B51] Karl T. Duffy L. Scimone A. Harvey R. P. Schofield P. R. (2007). Altered motor activity, exploration and anxiety-like in heterozygous neuregulin 1 mutant mice: implications for understanding schizophrenia. Genes Brain Behav. 6, 677–687 10.1111/j.1601-183X.2006.00298.x 17309661

[B52] Keri S. Kiss I. Seres I. Kelemen O. (2009). A polymorphism of the neuregulin 1 gene (SNP8NRG243177/rs6994992) affects reactivity to expressed emotion in schizophrenia. Am. J. Med. Genet. B Neuropsychiatr. Genet. 150B, 418–420 10.1002/ajmg.b.30812 18543275

[B53] Koenig J. I. Kirkpatrick B. Lee P. (2002). Glucocorticoid hormones and early brain development in schizophrenia. Neuropsychopharmacology 27, 309–318 10.1016/S0893-133X(01)00396-7 12093605

[B54] Krishnan V. Han M. H. Graham D. L. Berton O. Renthal W. Russo S. J. (2007). Molecular adaptations underlying susceptibility and resistance to social defeat in brain reward regions. Cell 131, 391–404 10.1016/j.cell.2007.09.018 17956738

[B55] Laarakker M. van Lith H. Ohl F. (2011). Behavioral characterization of A/J and C57BL/6J mice using a multidimensional test: association between blood plasma and brain magnesium-ion concentration with anxiety-like. Physiol. Behav. 102, 205–219 10.1016/j.physbeh.2010.10.019 21036185

[B56] Long L. E. Chesworth R. Arnold J. C. Karl T. (2010). A follow-up study: acute behavioural effects of Delta(9)-THC in female heterozygous neuregulin 1 transmembrane domain mutant mice. Psychopharmacology (Berl.) 211, 277–289 10.1007/s00213-010-1896-6 20571781

[B57] Long L. E. Chesworth R. Huang X. F. McGregor I. S. Arnold J. C. Karl T. (2013). Transmembrane domain Nrg1 mutant mice show altered susceptibility to the neurobehavioural actions of repeated THC exposure in adolescence. Int. J. Neuropsychopharmacol. 16, 163–175 10.1017/S1461145711001854 22226049

[B58] Long L. E. Chesworth R. Huang X. F. Wong A. Spiro A. McGregor I. S. (2012). Distinct neurobehavioural effects of cannabidiol in transmembrane domain neuregulin 1 mutant mice. PLoS ONE 7:e34129 10.1371/journal.pone.0034129 22509273 PMC3317922

[B59] Mackay-Sim A. Feron F. Eyles D. Burne T. McGrath J. (2004). Schizophrenia, vitamin D, and brain development. Int. Rev. Neurobiol. 59, 351–380 10.1016/S0074-7742(04)59014-1 15006495

[B60] Marenco S. Weinberger D. R. (2000). The neurodevelopmental hypothesis of schizophrenia: following a trail of evidence from cradle to grave. Dev. Psychopathol. 12, 501–527 10.1017/S0954579400003138 11014750

[B61] McGrath J. Saha S. Welham J. El Saadi O. MacCauley C. Chant D. (2004). A systematic review of the incidence of schizophrenia: the distribution of rates and the influence of sex, urbanicity, migrant status and methodology. BMC Med. 2:13 10.1186/1741-7015-2-13 15115547 PMC421751

[B62] McOmish C. E. Burrows E. Howard M. Scarr E. Kim D. Shin H. S. (2008). Phospholipase C-beta1 knockout mice exhibit endophenotypes modeling schizophrenia which are rescued by environmental enrichment and clozapine administration. Mol. Psychiatry 13, 661–672 10.1038/sj.mp.4002046 17667964

[B63] Mei L. Xiong W. C. (2008). Neuregulin 1 in neural development, synaptic plasticity and schizophrenia. Nat. Rev. Neurosci. 9, 437–452 10.1038/nrn2392 18478032 PMC2682371

[B64] Mohn A. R. Gainetdinov R. R. Caron M. G. Koller B. H. (1999). Mice with reduced NMDA receptor expression display behaviors related to schizophrenia. Cell 98, 427–436 10.1016/S0092-8674(00)81972-8 10481908

[B65] Munafo M. R. Thiselton D. L. Clark T. G. Flint J. (2006). Association of the NRG1 gene and schizophrenia: a meta-analysis. Mol. Psychiatry 11, 539–546 10.1038/sj.mp.4001817 16520822

[B66] Ng E. McGirr A. Wong A. H. Roder J. C. (2013). Using rodents to model schizophrenia and substance use comorbidity. Neurosci. Biobehav. Rev. 37, 896–910 10.1016/j.neubiorev.2013.03.025 23567519

[B67] Olsson I. A. Dahlborn K. (2002). Improving housing conditions for laboratory mice: a review of “environmental enrichment.” Lab. Anim. 36, 243–270 10.1258/002367702320162379 12144738

[B68] O'Tuathaigh C. M. Hryniewiecka M. Behan A. Tighe O. Coughlan C. Desbonnet L. (2010). Chronic adolescent exposure to Delta-9-tetrahydrocannabinol in COMT mutant mice: impact on psychosis-related and other phenotypes. Neuropsychopharmacology 35, 2262–2273 10.1038/npp.2010.100 20631688 PMC3055315

[B69] O'Tuathaigh C. M. O'Sullivan G. J. Kinsella A. Harvey R. P. Tighe O. Croke D. T. (2006). Sexually dimorphic changes in the exploratory and habituation profiles of heterozygous neuregulin-1 knockout mice. Neuroreport 17, 79–83 10.1097/01.wnr.0000192738.31029.0a 16361955

[B70] Owen M. J. Craddock N. O'Donovan M. C. (2005). Schizophrenia: genes at last? Trends Genet. 21, 518–525 10.1016/j.tig.2005.06.011 16009449

[B71] Ozaki M. Sasner M. Yano R. Lu H. S. Buonanno A. (1997). Neuregulin-beta induces expression of an NMDA-receptor subunit. Nature 390, 691–694 9414162 10.1038/37795

[B72] Paus T. Keshavan M. Giedd J. N. (2008). Why do many psychiatric disorders emerge during adolescence? Nat. Rev. Neurosci. 9, 947–957 19002191 10.1038/nrn2513 PMC2762785

[B73] Rampon C. Tang Y. P. Goodhouse J. Shimizu E. Kyin M. Tsien J. Z. (2000). Enrichment induces structural changes and recovery from nonspatial memory deficits in CA1 NMDAR1-knockout mice. Nat. Neurosci. 3, 238–244 10.1038/72945 10700255

[B74] Rapoport J. L. Addington A. M. Frangou S. Psych M. R. (2005). The neurodevelopmental model of schizophrenia: update 2005. Mol. Psychiatry 10, 434–449 10.1038/sj.mp.4001642 15700048

[B75] Romeo R. D. Bellani R. Karatsoreos I. N. Chhua N. Vernov M. Conrad C. D. (2006). Stress history and pubertal development interact to shape hypothalamic-pituitary-adrenal axis plasticity. Endocrinology 147, 1664–1674 10.1210/en.2005-1432 16410296

[B76] Roy V. Belzung C. Delarue C. Chapillon P. (2001). Environmental enrichment in BALB/c mice: effects in classical tests of anxiety-like and exposure to a predatory odor. Physiol. Behav. 74, 313–320 10.1016/S0031-9384(01)00561-3 11714494

[B77] Sanders A. R. Duan J. Levinson D. F. Shi J. He D. Hou C. (2008). No significant association of 14 candidate genes with schizophrenia in a large European ancestry sample: implications for psychiatric genetics. Am. J. Psychiatry 165, 497–506 10.1176/appi.ajp.2007.07101573 18198266

[B78] Smith D. R. Striplin C. D. Geller A. M. Mailman R. B. Drago J. Lawler C. P. (1998). Behavioural assessment of mice lacking D1A dopamine receptors. Neuroscience 86, 135–146 10.1016/S0306-4522(97)00608-8 9692749

[B79] Spear L. P. (2000). The adolescent brain and age-related behavioral manifestations. Neurosci. Biobehav. Rev. 24, 417–463 10.1016/S0149-7634(00)00014-2 10817843

[B80] Spires T. L. Hannan A. J. (2005). Nature, nurture and neurology: gene-environment interactions in neurodegenerative disease. FEBS anniversary prize lecture delivered on 27 June 2004 at the 29th FEBS congress in Warsaw. FEBS J. 272, 2347–2361 10.1111/j.1742-4658.2005.04677.x 15885086

[B81] Stefansson H. Sigurdsson E. Steinthorsdottir V. Bjornsdottir S. Sigmundsson T. Ghosh S. (2002). Neuregulin 1 and susceptibility to schizophrenia. Am. J. Hum. Genet. 71, 877–892 10.1086/342734 12145742 PMC378543

[B82] Stefansson H. Steinthorsdottir V. Thorgeirsson T. E. Gulcher J. R. Stefansson K. (2004). Neuregulin 1 and schizophrenia. Ann. Med. 36, 62–71 10.1080/07853890310017585 15000348

[B83] Taylor S. B. Markham J. A. Taylor A. R. Kanaskie B. Z. Koenig J. I. (2011a). Sex-specific neuroendocrine and behavioral phenotypes in hypomorphic Type II Neuregulin 1 rats. Behav. Brain Res. 224, 223–232 10.1016/j.bbr.2011.05.008 21620900 PMC3159843

[B84] Taylor S. B. Taylor A. R. Markham J. A. Geurts A. M. Kanaskie B. Z. Koenig J. I. (2011b). Disruption of the neuregulin 1 gene in the rat alters HPA axis activity and behavioral responses to environmental stimuli. Physiol. Behav. 104, 205–214 10.1016/j.physbeh.2010.11.015 21092742 PMC3081908

[B85] Taylor S. B. Taylor A. R. Koenig J. I. (2012). The interaction of disrupted Type II Neuregulin 1 and chronic adolescent stress on adult anxiety-like- and fear-related behaviors. Neuroscience. [Epub ahead of print]. 10.1016/j.neuroscience.2012.09.045 23022220 PMC3568180

[B86] Thompson J. L. Pogue-Geile M. F. Grace A. A. (2004). Developmental pathology, dopamine, and stress: a model for the age of onset of schizophrenia symptoms. Schizophr. Bull. 30, 875–900 10.1093/oxfordjournals.schbul.a007139 15954196

[B87] Ting A. K. Chen Y. Wen L. Yin D. M. Shen C. Tao Y. (2011). Neuregulin 1 promotes excitatory synapse development and function in GABAergic interneurons. J. Neurosci. 31, 15–25 10.1523/JNEUROSCI.2538-10.2011 21209185 PMC3078582

[B88] Tosato S. Dazzan P. Collier D. (2005). Association between the neuregulin 1 gene and schizophrenia: a systematic review. Schizophr. Bull. 31, 613–617 10.1093/schbul/sbi043 16081509

[B89] Trainor B. C. Pride M. C. Villalon Landeros R. Knoblauch N. W. Takahashi E. Y. Silva A. L. (2011). Sex differences in social interaction behavior following social defeat stress in the monogamous California mouse (*Peromyscus californicus*). PLoS ONE 6:e17405 10.1371/journal.pone.0017405 21364768 PMC3045459

[B90] Tsuang M. T. Bar J. L. Stone W. S. Faraone S. V. (2004). Gene-environment interactions in mental disorders. World Psychiatry 3, 73–83 16633461 PMC1414673

[B91] Tsuang M. T. Stone W. S. Faraone S. V. (2001). Genes, environment and schizophrenia. Br. J. Psychiatry Suppl. 40, s18–s24 10.1192/bjp.178.40.s18 11315219

[B92] Tucci V. Lad H. Parker A. Polley S. Brown S. Nolan P. (2006). Gene-environment interactions differentially affect mouse strain behavioral parameters. Mamm. Genome 17, 1113–1120 10.1007/s00335-006-0075-x 17091318

[B93] Valdar W. Solberg L. C. Gauguier D. Cookson W. O. Rawlins J. N. P. Mott R. (2006). Genetic and environmental effects on complex traits in mice. Genetics 174, 959–984 10.1534/genetics.106.060004 16888333 PMC1602068

[B94] van Dellen A. Blakemore C. Deacon R. York D. Hannan A. J. (2000). Delaying the onset of Huntington's in mice. Nature 404, 721–722 10.1038/35008142 10783874

[B95] van den Buuse M. Wischhof L. Lee R. X. Martin S. Karl T. (2009). Neuregulin 1 hypomorphic mutant mice: enhanced baseline locomotor activity but normal psychotropic drug-induced hyperlocomotion and prepulse inhibition regulation. Int. J. Neuropsychopharmacol. 12, 1383–1393 10.1017/S1461145709000388 19400983

[B96] van Os J. Kenis G. Rutten B. P. (2010). The environment and schizophrenia. Nature 468, 203–212 10.1038/nature09563 21068828

[B97] van Venrooij J. A. Fluitman S. B. Lijmer J. G. Kavelaars A. Heijnen C. J. Westenberg H. G. (2010). Impaired neuroendocrine and immune response to acute stress in medication-naive patients with a first episode of psychosis. Schizophr. Bull. 38, 272–279 10.1093/schbul/sbq062 20558533 PMC3283141

[B98] van Winkel R. Stefanis N. C. Myin-Germeys I. (2008). Psychosocial stress and psychosis. A review of the neurobiological mechanisms and the evidence for gene-stress interaction. Schizophr. Bull. 34, 1095–1105 10.1093/schbul/sbn101 18718885 PMC2632486

[B99] Walker E. Bollini A. M. (2002). Pubertal neurodevelopment and the emergence of psychotic symptoms. Schizophr. Res. 54, 17–23 10.1016/S0920-9964(01)00347-4 11853974

[B100] Walker E. Mittal V. Tessner K. (2008). Stress and the hypothalamic pituitary adrenal axis in the developmental course of schizophrenia. Annu. Rev. Clin. Psychol. 4, 189–216 10.1146/annurev.clinpsy.4.022007.141248 18370616

[B101] Walss-Bass C. Liu W. Lew D. F. Villegas R. Montero P. Dassori A. (2006). A novel missense mutation in the transmembrane domain of neuregulin 1 is associated with schizophrenia. Biol. Psychiatry 60, 548–553 10.1016/j.biopsych.2006.03.017 16730337

[B102] Weickert C. S. Hyde T. M. Lipska B. K. Herman M. M. Weinberger D. R. Kleinman J. E. (2003). Reduced brain-derived neurotrophic factor in prefrontal cortex of patients with schizophrenia. Mol. Psychiatry 8, 592–610 10.1038/sj.mp.4001308 12851636

[B103] Wong A. H. Van Tol H. H. (2003). Schizophrenia: from phenomenology to neurobiology. Neurosci. Biobehav. Rev. 27, 269–306 10.1016/S0149-7634(03)00035-6 12788337

[B104] Zammit S. Owen M. J. Evans J. Heron J. Lewis G. (2011). Cannabis, COMT and psychotic experiences. Br. J. Psychiatry 199, 380–385 10.1192/bjp.bp.111.091421 21947654

[B105] Zammit S. Owen M. J. Lewis G. (2010). Misconceptions about gene-environment interactions in psychiatry. Evid. Based Ment. Health 13, 65–68 10.1136/ebmh1056 20682811

[B106] Zhe D. Fang H. Yuxiu S. (2008). Expressions of hippocampal mineralocorticoid receptor (MR) and glucocorticoid receptor (GR) in the single-prolonged stress-rats. Acta Histochem. Cytochem. 41, 89–95 10.1267/ahc.08013 18787639 PMC2532603

[B107] Zhuang X. Oosting R. S. Jones S. R. Gainetdinov R. R. Miller G. W. Caron M. G. (2001). Hyperactivity and impaired response habituation in hyperdopaminergic mice. Proc. Natl. Acad. Sci. U.S.A. 98, 1982–1987 10.1073/pnas.98.4.1982 11172062 PMC29368

